# Datasets on process transesterification of binary blend of oil for fatty acid ethyl ester (FAEE) synthesized via the ethanolysis of heterogeneous doped catalyst

**DOI:** 10.1016/j.dib.2020.105905

**Published:** 2020-06-23

**Authors:** T.F. Adepoju, M.A. Ibeh, E.O. Babatunde, G.S. Abegunde, P.O. Adepoju, A.J. Asuquo, C.O. Osueke

**Affiliations:** a Chemical/Petrochemical Engineering Department, Akwa-Ibom State University, Ikot Akpaden, Mkpat Enin L.G.A., Akwa-Ibom State, P.M.B 1167, Uyo, Nigeria; b Agricultural and Biosystems Engineering Department, Faculty of Engineering, University of Ilorin, Kwara State, P.M.B 1515, Ilorin, Nigeria; c Chemical Engineering Department, Faculty of Engineering, University of Ilorin, Kwara State, P.M.B 1515, Ilorin, Nigeria; d Mechanical Engineering Department, College of Engineering, Landmark University, Omu Aran, Kwara State, P.M.B 1106, Ilorin, Nigeria

**Keywords:** API gravity ratio, Fatty acid ethyl ester (FAEE), Response surface methodology (RSM), Catalyst characterization, Physicochemical properties, Catalyst reusability test

## Abstract

The data employed the blend of waste used oil and beef tallow for the synthesis of fatty acid ethyl ester (FAEE) via ethanolysis of developed catalyst from calcined fermented cocoa pod husk powder (CFCPHP) doped with burnt cocoa pod husk powder (BCHP). Characterization of the developed doped catalyst (DDC) was carried out using FTIR, SEM, XRD, and BET adsorption analysis, while the basic strength of the DDC was tested through reusability test data. Mathematical optimization of the process condition was carried out through Box-Behnken Experimental Design (BBED) in 29 runs with variations in four variables as catalyst concentration (1.5 to 3.5 wt.%), reaction time (60 to 100 min), ethanol/oil molar ratio (EtOH/OMR) of 3 to 7, and reaction temperature (60 to 80 °C). The FAEE quality was ascertained by determining its fuel properties.

The data showed that the binary blend ratio of 42:58 of Waste Used oil: Beef Tallow oil (WUO: BTO) was obtained through API gravity ratio formulation. The developed doped catalyst (DDC) produced a high CaO-base of 84.30 (wt.%), with a high total basic site of 210 μmole.g^−1^ via BET and XRD analysis. The SEM analysis dataset showed non-uniform sizes, highly porous and crystalline sample, while the dataset on FTIR analysis data confirmed the presence of wagging and twisting CO_3_^2−^, the bending vibration of O—Ca-O, the sp^2^ of C = O, C = C, the sp of C  ≡  C and C  ≡  N, the bending structure of O—H, and the O=O, N ≡ O of Amines, and Amides. Based on the experiment, the maximum experimental yield of 97.80 (%wt.) at runs 7, and low yield of 89.50 (%wt.) at run 17 was obtained for FAEE. Mathematical optimization in 10 solutions predicted the FAEE yield of 97.7999 (%wt.) at the catalyst concentration of 3.10 (wt.%), the reaction time of 68.09 min, the EtOH/OMR of 3.01, and the reaction temperature of 72.21 °C. This data was validated in replicate, and the average mean value of FAEE was 97.68 (%wt.). Dataset on ANOVA and parametric analysis showed that the variable factors considered were significant at p-value <0.0001, with high R^2^ of 99.14%, R^2^-predicted of 98.32%, R^2^-adjusted of 98.28%, and adequate precision of 51.152, respectively. Catalyst reusability test data showed that the cycle number was stopped at the 5th cycle due to the decrease in catalyst basic strength. The produced FAEE dataset was within the recommended standard, and the data showed the developed doped catalyst successfully converted binary blend oil to FAEE, and the fermentation process increased the CaO-based conversion of DDC.

**Specifications Table**

**Table:** 

**Subject**	Material Science Engineering
**Specific subject area**	Renewable Energy
**Type of data**	Table, Chart, Figure
**How data were acquired**	Data on a binary mixture of the oil was acquired through the American Petroleum Institute (API) gravity computation of individual oil. Dataset on the physicochemical properties of the oil, the blended oil (BO), and the fatty acid ethyl ester (FAEE) were obtained through the **official methods of analyses of the Association of Official Analytical Chemists [**1**]. Data obtained** for a developed catalyst from the blended oil were obtained through catalysts calcination and characterization via Scanning electron microscopy (SEM), Fourier transforms infrared spectroscopy (FTIR), X-ray diffraction analysis (XRD), and Brunauer-Emmett-Teller (BET-adsorption) analysis. Process optimization of experimental data was obtained through Box Behnken Experimental Design (BBED). Multi-variable factors dataset levels were obtained through Analysis of Variance (ANOVA). The quality of the Fatty Acid Ethyl Ester (FAEE) synthesized was ascertained by comparing the dataset with biodiesel recommended standard.
	Data on catalyst potential and reusability was acquired through the catalyst reusability test.
**Data format**	Raw, Characterized, Synthesized, Analysed, Compared
**Parameters for data collection**	Waste used oil (WUO) was obtained from a local restaurant, in Eket, while beef tallow was obtained from an abattoir in Mkpat Enin L.G.A, Akwa Ibom State, Nigeria.
	The WUO was purified, heated, filtered, and allowed to cool at room temperature. The collected beef tallow was washed; centrifuged filtered and kept in a clean covered jar for further use.
	Cocoa pod husk was collected from a local Cocoa processing village in Ondo State. The pod husk was washed with distilled water, separated into samples A and B. Sample A was fermented, decanted, sun-dried, milled, calcined, while Sample B was sun-dried and burnt to ash. The two sample were sieved, and then mixed in ratio 1:1 to make Sample C. Process optimization of transesterification step was carried out by considering four variable factors: catalyst concentration (Ɛ1), reaction time (Ɛ2), ethanol/oil molar ratio (EtOH/OMR (Ɛ3)), and reaction temperature (Ɛ4), respectively with FAEE as the response variable. Catalyst reusability test was carried out via catalyst recyclability.
**Description of data collection**	The blend of oil was obtained by determining the specific gravity of constituent oil, and then estimating the API gravity using an appropriate equation.
	Fermented cocoa pod husk powder was calcined in a furnace for 4 h at 750 °C to obtain calcined fermented cocoa pod husk powder (CFCPHP), which was doped with burnt cocoa pod husk ash powder (BCPHAP) in the open air to obtain a developed doped catalyst (DDC) used for biodiesel production of blended oil.
	Since the blended oil properties are within the low acid value [2,3,4], low viscosity, and moderate density [5,6,7] for successful transesterification, a one-step process was used by employing the method earlier reported by [7], but with little modification. Catalyst reusability test was carried out through catalyst recyclability, purification, and analysis. The test was stopped at the 5th cycle, while FAEE quality was determined and compared with [8] and [9] recommended standard.
**Data source location**	Akwa Ibom State University, Akwa Ibom State, Nigeria, Africa.
**Data accessibility**	Data with the article

**Value of the Data**

• Data are useful because they provide an analytical method on the blend of two or more oil, and also provide the way by which more waste can be harness for catalytic formation.
• Data can be beneficial to researchers in the field of Chemical and agro-allied industries involved in the use of CaO-base catalysts as feedstock for production.
• Data can be used as a basis for biodiesel synthesis via various catalysts derived from agricultural waste under the same experimental conditions.
• The data potential will make both short and long term impact on society in the field of renewable and sustainable energy.
• Dataset is the first report on the development of CaO-base catalyst derived from calcined fermented cocoa pod husk powder doped with burnt cocoa pod husk powder

## 1 Data description

**Fig. 1:**
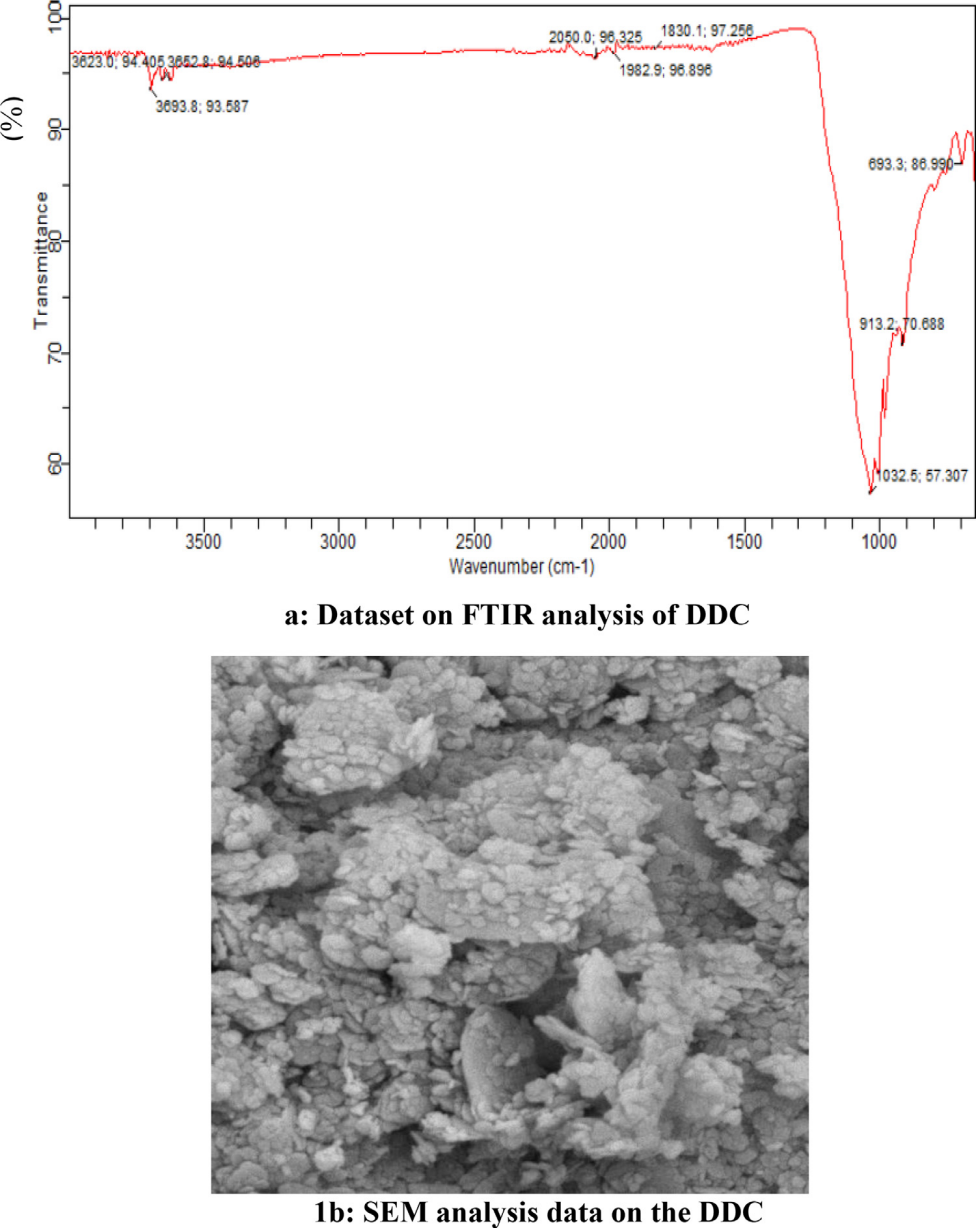
a: Dataset on FTIR analysis of DDC, Fig. 1b: SEM analysis data on the DDC.

**Fig. 2:**
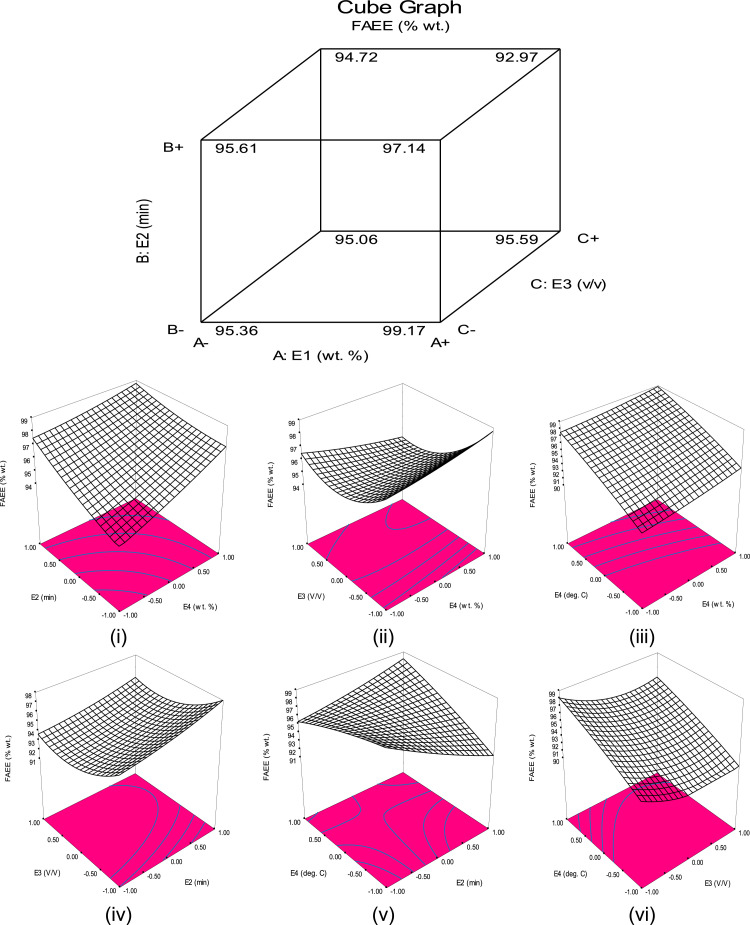
a: The cubic data plot. Fig. 2b (i-vi): Three-dimensional plots showing the interactions of the variables.

**Fig. 3:**
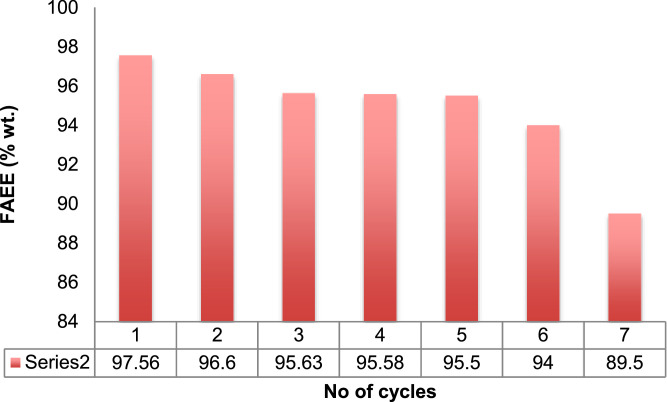
Catalyst reusability test data.

Dataset on the physicochemical properties of the oil and its blend are presented in Table 1. The estimated ratio through API gravity was also included in the table. Fig. 1a which showed the sinusoidal 8 cm^−1^ resolution of the data plot at different wavelengths and transmittance of FTIR analysis of the doped catalyst is displayed, while Fig. 1b which showed the size, the nature, the porosity, and the spongy nature of the doped catalyst area as displayed by SEM image. Table 2 showed the functional groups present in the doped catalyst further explained by the FTIR analysis. Displayed in Table 3 are the dataset on basicity, the porous volume, the basic density site, the surface bet, and the total basic density as produced by BET adsorption analysis. The table also displayed the data produced by XRD analysis which showed the composition of CaO-based catalyst present in the doped catalyst. Table 4a showed the data on experimental set of three-level and the four factors used for the experimental design, while Table 4b showed the dataset on the yield of FAEE, the predicted value, the residual, and the coded levels of the four variables used for experimental data as generated by BBED, Table 4c showed the ANOVA dataset on level of significance of the variables and the parametric values, and Table 4d displayed the dataset on 10 predicted solutions. The second-order model equation that correlates the variables considered for transesterification and the response FAEE was presented in Eq. (1). Fig. 2a observed the cubic data plot of E_1_, E_2_, and E_3_, while keeping the E_4_ constant at 72.21 °C on the response (FAEE), while Fig. 2b(i-vi) indicated the three-dimensional plots which illustrated the interactions among the variables considered for process optimization. The plots of catalyst reusability test data are presented in Fig. 3 at catalyst concentration of 2.5 (wt.%), while the quality of the FAEE data as compared with biodiesel standard are presented in Table 5. The raw dataset is as presented in the supplementary data file.

(1) $$\begin{array}{ccc} \text{FAEE}\;\left(\%\;\text{wt}.\right) & = & +93.66+0.55X_{1}-1.12X_{2}-1.12X_{3}+0.53X_{4}+0.16X_{1}^{2}+0.27X_{2}^{2}+1.51X_{3}^{2}\\ & & -\;0.30X_{4}^{2}\;-0.57X_{1}X_{2}-0.82X_{1}X_{3\;}-0.17X_{1}X_{4}-0.15X_{2}X_{3}+2.37X_{2}X_{4}-0.0025X_{3}X_{4} \end{array}$$

**Table 1:** Data on the properties of the oil.

Properties	WUO	BTO	BO	Total API gravity
Moisture content (%)	0.01	0.01	0.008^a^	
Viscosity @ 40 °C/ (mm^2^/s)	1.20	2.21	1.97^a^	
Acid value(mg KOH/g oil)	0.94	1.20	1.04^a^	
% Free Fatty Acid (FFA)	0.47	0.60	0.52^a^	
Peroxide value (meq O_2_/kg oil)	6.24	8.40	7.43^a^	
Saponification value (mg KOH/g oil)	156.23	186.74	172.32^a^	
Iodine value (g I_2_/100 g oil)	54.82	68.84	64.47^a^	
Specific gravity	0.88	0.82	0.84^a^	
API gravity	29.30	41.06	36.95^a^	70.36^b^
Blend ratio	**41.64**	**58.36**		
Simplex blend ratio	**42**	**58**		

Here, *a* = after blend, *b* = before blend, BO = blended oil.

**Table 2:** FTIR data on DDC spectrum.

SN	Wavelength (cm^−1^)	Transmittance (%)	Bonds and Functional groups
1	693.31 to 913.2	86.990 to 70.688	C-I, C-Br, C—Cl, N—H, and waging and twisting CO_3_^2-^
2	1032.5 to 1830.1	57.307 to 97.256	C—C, C=C, C=N, and bending vibration of O—Ca-O
3	1982.9 to 2050.0	96.896 to 96.325	C=O, CHO, C=C, C ≡ C, C ≡ N, and O—H
4	3623.8 to 3693.8	94.506 to 93.587	O-H bending structure, O=O, N ≡ O, Amine, and Amide

**Table 3:** Dataset on BET and XRD analysis of catalysts showing the catalysts nature.

Catalysts	N_2_-AA (m^2^g^−1^)	TPV (cm^3^g^−1^)	%CaO	BS (μmole.g^−1^)		TBS	BSD (μmole.m^−2^)	FAEE (%wt.)
				400<BS<550	550<BS≤750			
CFCPHP	1.10	0.0035	78.45	24	150	174	158.18	90.56
BCPHP	1.20	0.0035	65.40	16	146	162	135.00	87.40
DDC	1.30	0.0035	84.30	28	184	210	161.54	99.85

N_2_-AA= nitrogen adsorption analysis, TPV = Total pore volume, BS = Basic site, TBS = Total basic site, BSD = Basic site density.

**Table 4a:** Experimental dataset for transesterification.

Variables	Units	Symbol	Levels		
			−1	0	1
Catalyst concentration	(wt.)	Ɛ_1_	1.5	2.5	3.5
Reaction time	(min)	Ɛ_2_	60	80	100
EtOH/OMR	(v/v)	Ɛ_3_	3	5	7
Reaction temperature	( °C)	Ɛ_4_	60	70	80

**Table 4b:** The experimental, the predicted, and the residual dataset on FAEE.

Runs	E_1_ (wt.%)	E_2_ (min)	E_3_ (v/v)	E_4_ (°C)	FAEE (% wt.)	PFAEE (% wt.)	R (% wt.)
1	0	0	0	0	93.60	94.08	−0.080
2	1	0	0	1	93.70	96.33	−0.065
3	0	1	0	1	93.00	92.99	0.032
4	0	0	−1	1	96.50	92.95	0.047
5	−1	0	1	0	94.50	95.46	0.045
6	0	0	0	0	94.00	93.22	−0.013
**7**	**1**	**0**	**−1**	**0**	**97.80**	**96.52**	**−0.020**
8	1	0	0	−1	93.70	94.28	−0.078
9	1	1	0	0	93.00	92.27	0.065
10	−1	0	−1	0	95.00	93.71	−0.005417
11	0	0	1	−1	93.21	93.66	0.040
12	0	−1	−1	0	97.56	94.43	−0.030
13	−1	0	0	−1	92.33	97.53	0.033
14	0	0	−1	−1	95.50	95.59	0.040
15	0	0	0	0	93.80	95.59	−0.005417
16	0	0	1	1	94.20	93.06	0.001250
**17**	**0**	**1**	**0**	**−1**	**89.50**	**95.08**	**−0.076**
18	1	0	0	1	94.40	97.82	−0.021
19	0	0	0	0	93.00	94.48	0.020
20	1	0	1	0	94.02	93.94	0.075
21	1	−1	0	0	96.26	96.59	0.014
22	0	1	1	0	93.06	89.60	−0.10
23	−1	1	0	0	93.02	92.90	0.10
24	0	−1	1	0	95.58	95.41	−0.015
25	0	1	−1	0	95.63	93.66	0.34
26	0	1	0	1	95.40	93.66	−0.66
27	0	−1	0	−1	96.60	93.66	0.14
28	0	0	0	0	93.90	93.66	0.24
29	−1	−1	0	0	94.00	93.66	−0.060

PFAEE = predicted free fatty acid ethyl ester, *R* = residual.

**Table 4c:** ANOVA and parametric data fits statistics.

**Source**	**Sum of Squares**	**df**	**Mean Square**	**F Value**	**Prob > *F***	**Outcomes**
Model	80.95	14	5.78	115.91	< 0.0001	Significant
E_1_	3.66	1	3.66	73.43	< 0.0001	Significant
E_2_	14.94	1	14.94	299.51	< 0.0001	Significant
E_3_	15.01	1	15.01	300.85	< 0.0001	Significant
E_4_	3.37	1	3.37	67.57	< 0.0001	Significant
E_1_^2^	0.16	1	0.16	3.26	0.0925	Not significant
E_2_^2^	0.47	1	0.47	9.36	0.0085	Significant
E_3_^2^	14.83	1	14.83	297.30	< 0.0001	Significant
E_4_^2^	0.60	1	0.60	11.93	0.0039	Significant
E_1_E_2_	1.30	1	1.30	26.05	0.0002	Significant
E_1_E_3_	2.69	1	2.69	53.92	< 0.0001	Significant
E_1_E_4_	0.11	1	0.11	2.25	0.1559	Not significant
E_2_E_3_	0.087	1	0.087	1.74	0.2078	Not significant
E_2_E_4_	22.56	1	22.56	452.29	< 0.0001	
E_3_E_4_	0.000025	1	0.000025	0.0005012	0.9825	Not significant
Residual	0.70	14	0.050	–	–	Significant
Lack of Fit	0.066	10	0.006639	0.042	1.0000	Not significant
Pure Error	0.63	4	0.16	–	–	Not significant
Cor Total	80.95	14	5.78	115.91	< 0.0001	Significant
Std. Dev.	0.22		R-Squared		0.9914	
Mean	94.34		Adj R-Squared		0.9828	
C.V.	0.24		Pred R-Squared		0.9832	
PRESS	1.37		Adeq Precision		51.152

**Table 4d:** Dataset on 10 predicted solutions for experimental validation.

No	E_1_ (wt.%)	E_2_ (min)	E_3_ (v/v)	E_4_ (deg. C)	FAEE (% wt.)	
1	3.10	68.09	3.01	72.21	97.7999	Selected
2	3.49	60.81	4.72	62.22	97.8000	
3	3.46	60.29	4.60	63.52	97.8001	
4	2.42	99.65	3.03	78.91	97.8002	
5	2.48	62.50	3.08	65.36	97.8000	
6	3.45	73.27	3.01	76.94	97.7999	
7	2.38	96.76	3.00	79.99	97.7999	
8	2.00	99.91	3.01	79.63	97.8002	
9	3.03	96.97	3.12	78.62	97.7999	
10	3.5	60.05	7.00	60.76	97.6810

**Table 5:** Dataset on quality of blended oil and FAEE as compared with biodiesel standard.

Parameter	Blended oil	FAEE	[8]	[9]
Colour@ 27 oC	Light yellowish	Brownish	–	–
State @ room temp	Liquid	Liquid	Liquid	Liquid
Density (kg/m^3^) @ 25 °C	870	860	–	860–900
Viscosity @ 40 °C/ (mm^2^/s)	1.97	1.90	1.9–6.0	3.5–5.0
Moisture content (%)	0.008	<0.03	<0.03	0.02
%FFA (as oleic acid)	0.52	0.14	0.40 max	0.25 max
Acid value (mg KOH/g oil)	1.04	0.07	0.80 max	0.50 max
Iodine value (g I_2_/100 g oil)	64.47	60.72	–	120 max
Saponification value (mg KOH/g oil)	172.32	152.24	236.66–253.04	–
Peroxide value (meq O_2_/kg oil)	7.43	6.21	–	12.85
HHV (MJ/kg)	41.40	42.28	–	–
Cetane number	63.47	68.49	57 min	51 min
API gravity	31.14	33.03	30–42	–
Diesel index	61.46	68.01	50.4 min	–

## 2 Experimental design, materials, and methods

The WUO was heated in an opened container at 100 ^o^C on a hot plate for 30 min and was allowed to cool to room temperature, then filtered to remove dirt. The cleaned oil was then stored in a covered jar for further processing. The collected beef tallow was washed in a container with 500 ml of Na_2_CO_3_ (1 mol/l) and stirred for 30 min mechanically. The mixture was centrifuged at 3500 rpm for 10 min at the temperature of 25 ^o^C using a propylene tube. The supernatant was separated by filtration, and 40 g of anhydrous Na_2_SO_4_ was added, stirred for 8 min, and was centrifuged again for 6 min at the temperature of 25 ^o^C [2]. The pure beef tallow oil (PBTO) obtained was kept in a clean covered jar for further use.

The cocoa pod husk was washed with distilled water thrice and allowed to dry at room temperature for 24 h. After 24 h, the washed pod husks now free of water were divided into two-sample A and B. Sample B was submerged in distilled water and anaerobically fermented for 10 days, while sample C was sun-dried. After 10 days, the fermented sample B was decanted off the water, oven-dried at 120 ^o^C until a constant weight was achieved, and then milled into powder before being calcined in a furnace at 750 ^o^C for 4 h, while sample A was sun-dried to constant weight, and burnt to ash in the open air. The two powders were made into smaller particle sizes (0.30 mm) by sieving. The calcined fermented cocoa pod husk powder (CFCPHP) was doped with burnt cocoa pod husk powder in the same ratio to obtain the developed doped catalyst. The developed doped catalyst was characterized using SEM to observe the surface morphology of the samples, XRD has been performed using Cu-Kα radiation source accelerated at 10 mA and 30 kV in the wide range of 2θ (10° <2θ <80°) at a speed of 2.5 ^o^/min and to verify the elemental analysis of and the quantitative composition of the samples. FTIR was used at a resolution of 8 cm^−1^ to confirm the presence of functional groups and verify the presence of characteristic absorption bands of major elements present within the crystal powder structures. The pore volume, surface area, basic density site, and the total basic density were examined using BET isothermal adsorption and Hammett indicator.

For oil blend, the major key parameters are the oil viscosity and the American Petroleum Institute gravity (API gravity) ratio. From the physicochemical properties of the oil, the API gravity of the oil was computed using Eqn. (2).

(2) $$\forall =\frac{131.5\left(1.076-\partial \right)}{\partial }$$

Here ∀ is the API gravity, ∂ the specific gravity of the oil.

Therefore, the API gravity of the oils was summed up to obtain the total API gravity (Eq. (3)), and the blend ratio (BR) was computed by dividing the API gravity of individual oil with the total API gravity (Eq. (4)). The blended oil (BO) was heated at 40 ^o^C on a magnetic stirrer for a homogeneous phase.

(3) $$\forall_{T}\;=\;\forall_{WUO}+\forall_{BTO}$$

(4) $$BR=\left(\frac{\forall_{WUO}\;}{\forall_{T}}X100:\;\frac{\forall_{BTO}\;}{\forall_{T}}X100\right)$$

∀_*T*_ represents the total API gravity. ∀_*WUO*_ and ∀_*BTO*_ are the API gravity of Waste used oil and Beef tallow oil. BR is the blended ratio. Properties of the oil and the blended oil were further determined using [1].

FAEE synthesis was carried out by the ethanolysis of CaO-based catalyst derived from the doped catalyst. The reaction process took place in a three-necked-reactor, 120 ml of the blended oil was preheated on a heating mantle for 70 min at 80 °C.

1.5 (wt.%) of the catalyst was added to 40 ml of ethanol in a 250 ml dried, clean flask to obtain EtOH/OMR of 1:3. The mixture was placed on a shaker for 15 min and then added to the preheated oil. Two layers were observed, the ethanol-catalyst layer and the oil layer. The stirrer was inserted into the mixture and the reaction was monitored at 60 ^o^C for 60 min. At the end of the reaction, the insoluble catalyst was separated through decantation, and the remained products (ethanol-biodiesel) was separated by gravity in a separating funnel. The obtained fatty acid ethyl ester (biodiesel) contained adherent catalyst (leached catalyst), which was removed by washing with hot methanolic sodium carbonate (1.0 g Na_2_CO_3_ dissolved in 20 ml methanol), and was well stirred. The washed mixture was filtered, and the filtrate-FAEE was washed with distilled water twice before water-diesel separation through gravity settling. The water wet-FAEE was dried over anhydrous disodium sulfate (Na_2_SO_4_), before separation by decantation to obtain pure biodiesel (fatty acid ethyl ester: FAEE). The residual catalyst filtered was collected for reuse but was first purified. This process was conducted based on a number of experimental runs.

For a linear correlation and proper experimental design, four-factor-three-level variables were considered for experimental design. A BBED was used under response surface methodology (RSM), a total of 29 experimental runs obtained through different combinations and was performed.

Optimization of process condition of FAEE synthesized from blended oil was carried out through ANOVA, the probability value (p-value), the factor value (f-value), the degree of freedom (df), and the variance inflation factor (VIF), respectively. Linear regression parameters were obtained through evaluation of the coefficient of determination the predicted coefficient of determination, the adjusted coefficient of determination, and the adequate precision (Adeq. Prec.) to confirm the model suitability. Meanwhile, to establish the relationship between interactive variables and the FAEE, three dimensional-contour plots were used [10]. The second-order polynomial model equation that further explains the relationship between FAEE yield and the independent variable factors is mathematically expressed as Eq. (5).

(5) $$FAEE\;\left(\%\;wt.\right)=P_{0}+\sum_{i=1}^{k}P_{i}\;\varepsilon_{i}+\sum_{i=1}^{k}P_{ii}\varepsilon_{i}^{2}+\sum_{i<j}^{k}P_{ij}\varepsilon_{i}\varepsilon_{j}+R$$

Where FAEE is the response in percentage, *P*_0_ is the intercept, *P_i_* is the linear coefficient, *P_ii_* is the interaction coefficient, *P_ij_* is the quadratic coefficient terms, ɛ_*i*_ and ɛ_*j*_ are the four factors and *R* is the residual error.

The recovered catalyst was tested for its efficiency by carrying out the reusability test but was purified before reuses. The recovered catalyst was purified through the method adopted by [2] with little modification- the recovered catalyst was washed with methanol to remove the impurity at the surface of catalyst that occurred during the production of FAEE. The washed catalyst with methanol was centrifuged in an inbuilt heating vacuum centrifuge operated at 3500 rpm, and separated by decantation. The wet catalyst was oven-dried at 100 ^o^C for 1 h to be free of methanol and then cooled to room temperature before being reused. The reusability test stopped at 6th and 7th cycles due to catalyst concentration degeneration.

Properties of the FAEE were determined and compared with [8] and [9] recommended standards to ascertain its suitability as a replacement for conventional fuel in an internal combustion engine (I.C. engine). Details of raw dataset on the experimental design, the modeling equation, the predicted dataset, the standard error plots, the contour plots, the three-dimensional plots in these datasest are as presented in the supplementary file.

## Declaration of Competing Interest

The authors declare that they have no competing financial interests or personal relationships which have, or could be perceived to have, influenced the work reported in this article.

This research work received no financial support from any financial Institution or government organization.

## Appendix Supplementary materials

Image, application 1

Image, application 2
